# Passive Eruption of a Soft-Tissue-Impacted Maxillary Canine Following Diode Laser Exposure: A Case Report

**DOI:** 10.7759/cureus.85526

**Published:** 2025-06-07

**Authors:** Gabrielle Tomeo, Carter E Bedinghaus, James Morrish, Jeung Woon Lee

**Affiliations:** 1 Dentistry, Lake Erie College of Osteopathic Medicine (LECOM) School of Dental Medicine, Bradenton, USA; 2 Dentistry, Lake Erie College of Osteopathic Medicine, Bradenton, USA

**Keywords:** adolescent orthodontics, canine, diode laser, impaction, passive dental eruption, soft tissue

## Abstract

Maxillary canine impactions are rather common and observed more frequently in female than male children. Typically, osseous obstruction is thought to be the cause of canine impaction, with soft tissue rarely considered a primary cause. This case report presents a 14-year-and-10-month-old female with a maxillary right canine impacted solely by soft tissue. Notably, the primary canine had exfoliated naturally and on time, yet the permanent successor failed to erupt. While timely exfoliation of the primary canine typically facilitates the proper eruption path of the permanent canine, this case underscores that even in the absence of hard-tissue obstruction, dense or fibrotic soft tissue alone may be sufficient to hinder eruption. Following diode laser exposure of the gingival tissue and passive space maintenance with a retainer, the canine passively erupted 3 mm over three months without the aid of brackets, chains, or orthodontic traction. This case challenges the traditional belief that soft tissue alone cannot cause impaction, and demonstrates that, in suitable cases, the diode laser intervention along with passive monitoring can be utilized as a minimally invasive alternative.

## Introduction

Impacted maxillary canines occur in approximately 1-3% of the population and are second only to third molars in frequency of impaction [1]. The maxillary canine plays a critical role in the maintenance of facial symmetry, arch integrity, esthetics, and proper occlusal guidance. Due to their long root structure and strategic position in the dental arch, their successful eruption is essential not only for functional occlusion but also for the long-term stability of orthodontic treatment outcomes [2].

Impactions are more prevalent in females, with a female-to-male ratio of approximately 2:1 [1,3]. They tend to occur more frequently in the maxilla than in the mandible and are commonly located palatally (in 85% of cases) rather than buccally [4,5]. The etiology of impaction is multifactorial and can be broadly categorized into genetic and environmental causes. Hereditary factors include familial patterns of ectopic eruption, dental anomalies such as peg laterals or missing lateral incisors, and discrepancies in tooth-to-arch size ratios [3,6]. Environmental contributors include retained deciduous canines, early loss of primary teeth, crowding, or trauma to the anterior maxilla during developmental stages [7].

The consequences of canine impaction are wide-ranging. If left untreated, an impacted canine may cause resorption of adjacent roots, particularly the lateral incisors, disrupt the midline or arch form, and compromise both smile esthetics and masticatory function [8,9]. Phonetic changes, especially involving sibilant sounds, can occur due to altered incisor or canine position. Psychosocial concerns may also arise from asymmetry or spacing, particularly in adolescent patients.

Traditionally, the diagnosis of impaction is made through clinical examination, supplemented with radiographs and, more recently, cone-beam computed tomography (CBCT) for the precise three-dimensional assessment of the tooth’s position [10]. Standard treatment modalities include surgical exposure (either open or closed technique), followed by orthodontic traction using bonded attachments, elastics, chains, or auxiliary devices such as TADs (temporary anchorage devices) [11]. In some cases, extraction of the impacted canine and prosthetic replacement may be necessary if alignment is deemed unfeasible.

While these conventional methods are effective, they are often invasive, time-consuming, and uncomfortable for the patient. Recently, diode laser exposure has emerged as a minimally invasive alternative for soft tissue removal, offering advantages such as hemostasis, reduced pain, and shorter healing times [12-14]. However, literature documenting passive eruption following diode laser exposure alone, without mechanical traction, remains scarce [15].

## Case presentation

**Figure 1:**
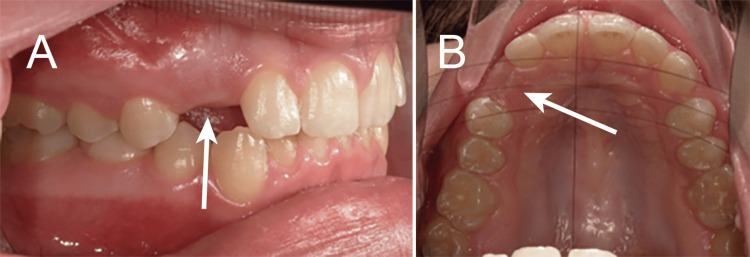
Unerupted right maxillary canine (#6) in a 14-year-and-10-month-old patient (arrows: A, facial view; B, occlusal view).

A 14-year-and-10-month-old Caucasian female presented to the orthodontic clinic at Lake Erie College of Osteopathic Medicine (LECOM) School of Dental Medicine seeking a second opinion regarding her orthodontic treatment, with specific concern about the delayed eruption of her upper right canine. During the review of her medical, dental, and family history, it was noted that she was congenitally missing her mandibular second permanent pre-molars along with the presence of retained primary second molars, a finding also reported in both her mother and grandmother, suggesting a possibly hereditary component [3,16]. Intraoral examination revealed healthy soft and hard tissues, with the exception of an unerupted maxillary right canine and congenitally missing mandibular first permanent molars (Figure 1).

**Figure 2:**
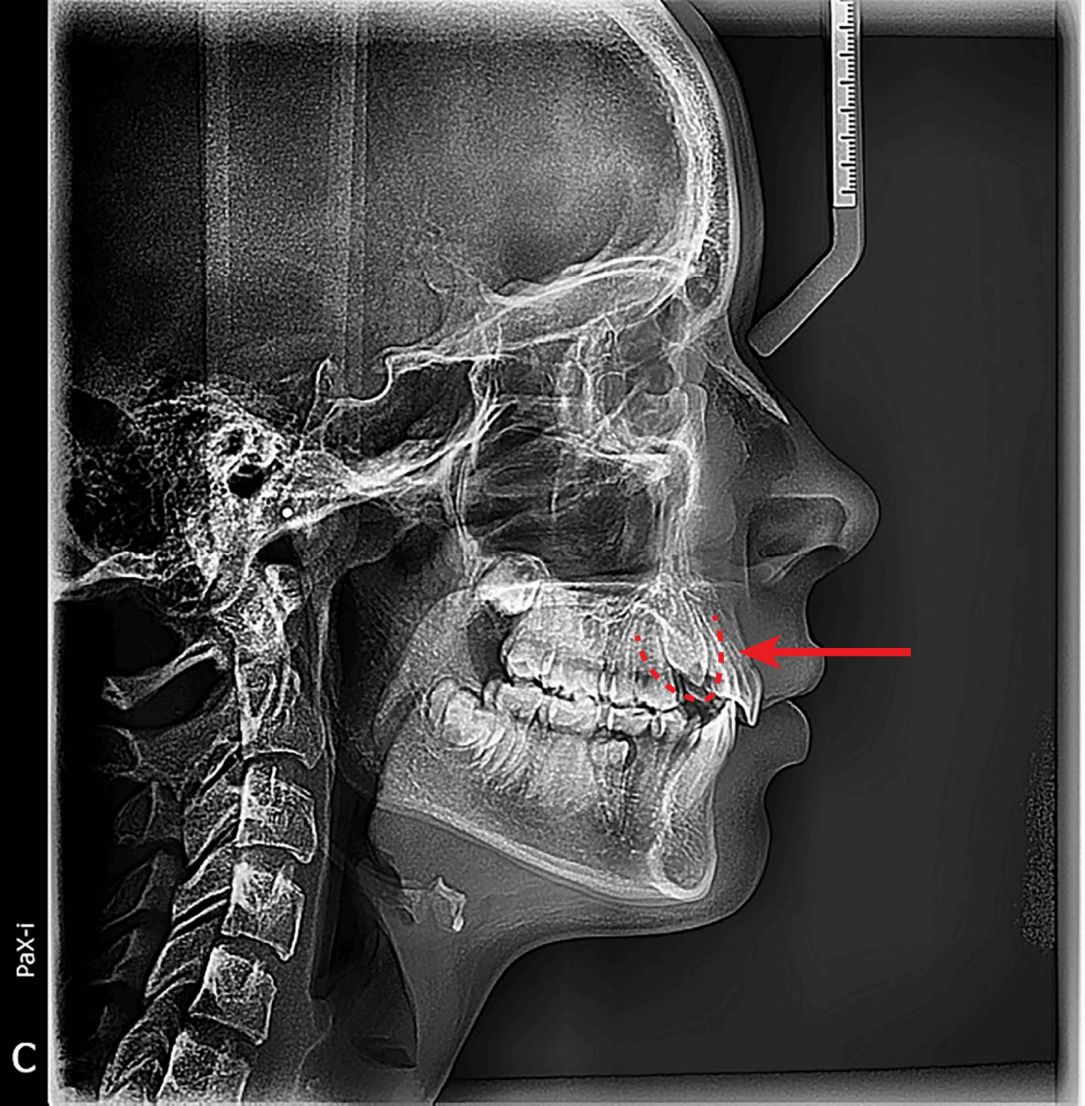
Cephalometric radiograph showing soft-tissue impaction in a 14-year-and-10-month-old patient (arrow).

A pre-eruptive bulge corresponding to tooth #6 was palpable on the buccal aspect of the alveolar ridge and was firm in consistency, suggestive of an underlying impacted canine. Radiographic evaluation seemed to suggest the presence of tooth #6 with no osseous coverage of the crown (Figure 2).

**Figure 3:**
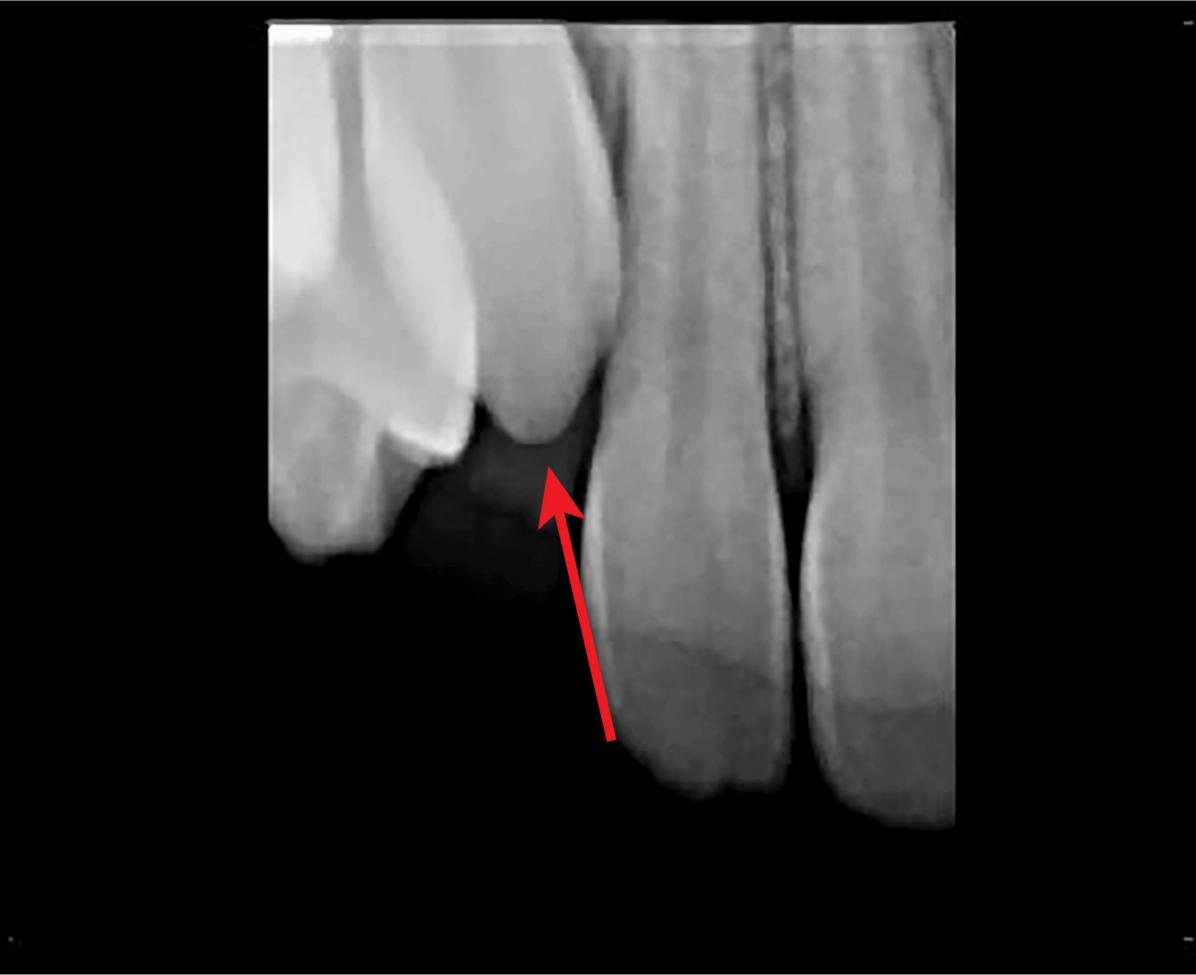
Initial periapical radiograph showing position #6 in a 14-year-and-10-month-old patient (arrow).

A periapical image indicated that tooth #6 was present and positioned near the alveolar crest (Figure 3).

**Figure 4:**
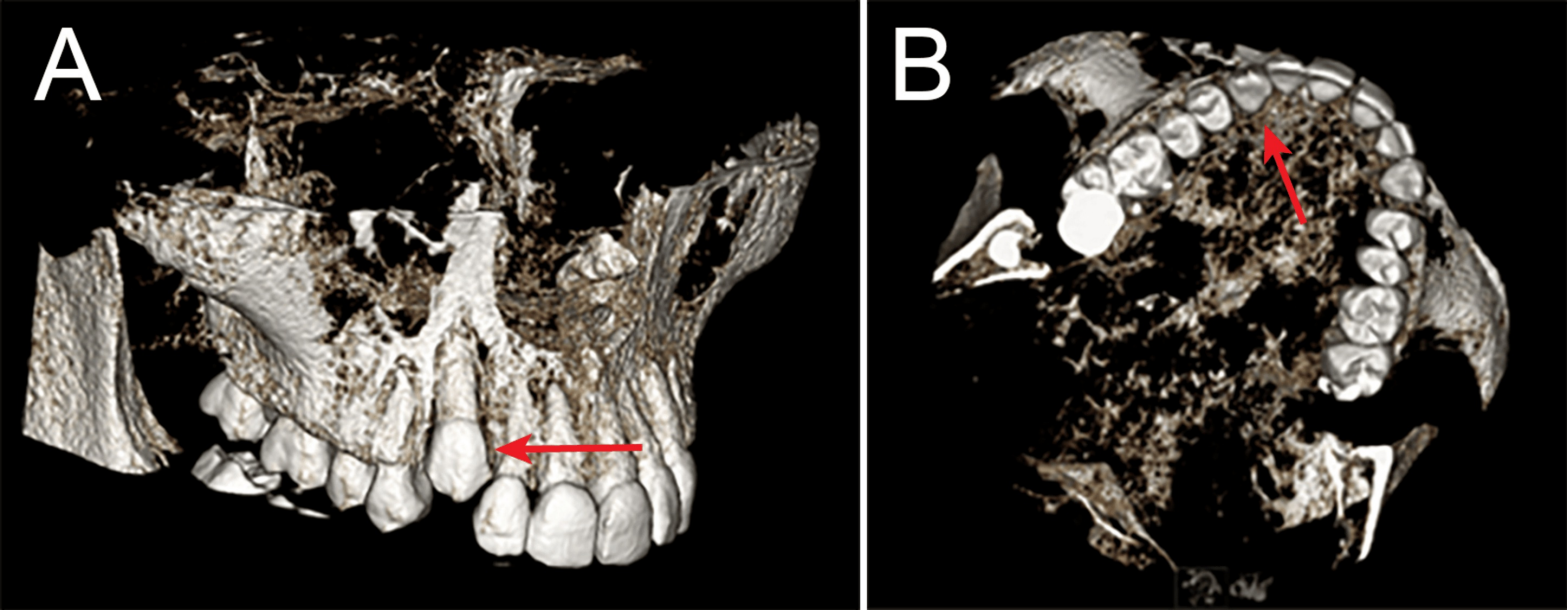
A: CBCT facial slice taken at 14 years and 10 months of age, demonstrating the absence of hard tissue around the crown of tooth #6 (arrow). B: CBCT occlusal slice also demonstrating the absence of hard tissue around the crown of tooth #6 (arrow). CBCT, cone-beam computed tomography

To further assess its exact location and orientation, a CBCT was obtained, along with an intraoral diagnostic scan. Evaluation by the periodontal department confirmed that there was soft tissue-based impaction, with no significant bony coverage. Additionally, sufficient space was available in the arch to accommodate natural eruptions (Figure 4).

Treatment 

Given the patient’s consistently positive behavior, reflected by a (+,+) Frankl behavior rating, and the family’s preference for a minimally invasive approach, the treatment was planned without the need for general anesthesia. The proposed plan involved diode laser-assisted exposure of tooth #6 without the use of orthodontic auxiliaries such as TADs or chain attachments. Passive monitoring of the eruption would follow, with space maintenance achieved through a clear Essix retainer.

Following standard protocol, one carpule of 4% articaine 1:100,000 mg epinephrine was administered via local infiltration around tooth #6. Salivation was managed with a saliva ejector. 

**Figure 5:**
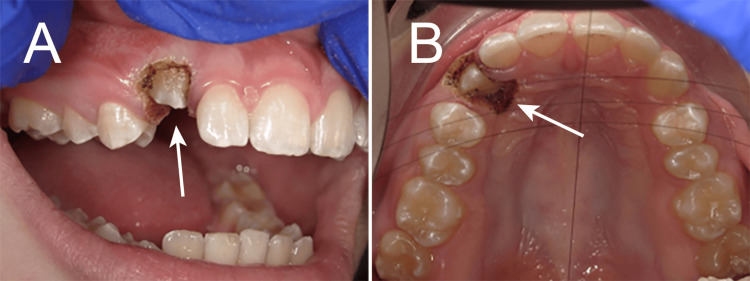
Intraoperative photograph of diode laser exposure of tooth #6 at 15 years and one month of age (arrow; A: facial view; B: occlusal view).

Clinical and radiographic evaluation served as the basis for the treatment plan. A diode laser was then used to excise the keratinized gingiva overlying the crown, at the time of the exposure the female was 15 years and one month of age. No osseous removal was needed. The canine was exposed and allowed to passively erupt (Figure 5).

Following the procedure, the area was thoroughly irrigated, and a clear retainer was delivered to be worn 20-22 hours per day to maintain space between teeth #5 and #7. The patient was informed to consume a soft diet and maintain oral hygiene.

**Figure 6:**
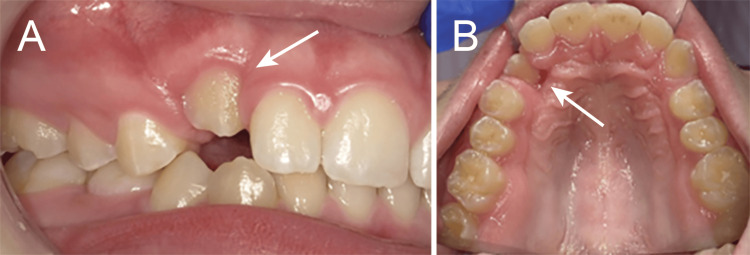
Clinical photograph showing 1 mm of eruption one month after laser exposure at 15 years and two months of age (arrow; A: facial view; B: occlusal view).

At the one-month follow-up, approximately 1 mm of the passive eruption of tooth #6 was observed. The surrounding gingival tissue appeared healthy, and the patient reported no discomfort (Figure 6).

**Figure 7:**
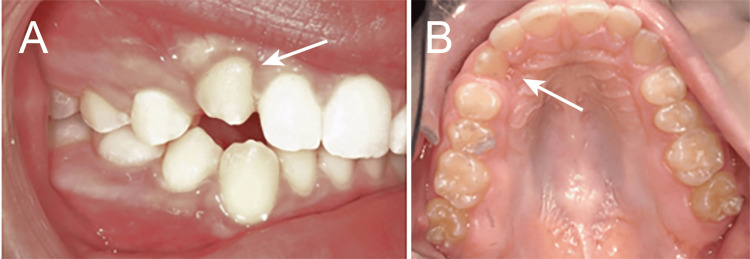
Three months after laser treatment, the canine at 15 years and three months of age exhibited 3 mm of eruption (arrow; A: facial view; B: occlusal view).

By the three-month follow-up, a total of approximately 3 mm of passive eruption of tooth #6 had occurred. The crown continued to progress into the arch without the application of any active orthodontic forces (Figure 7). Once an additional 2-3 mm of eruption occurs, clear aligner therapy is planned to finalize the tooth position of #6.

## Discussion

This case highlights a rarely reported phenomenon: tooth impaction caused exclusively by soft tissue. The conventional understanding of maxillary canine impactions emphasizes hard tissue barriers, such as adjacent teeth, dense cortical bone, or an abnormal eruption path, as the primary etiologies [1,17]. As a result, treatment protocols are typically surgical and mechanical, involving flap surgery, osseous removal, and orthodontic traction to facilitate eruption.

However, in this patient, radiographic and clinical findings confirmed the complete absence of bony coverage over tooth #6, with the crown positioned superficially and aligned favorably within the arch. Despite this, spontaneous eruption did not occur. Only after diode laser-assisted removal of the keratinized gingiva did the tooth begin to erupt passively, ultimately progressing 3 mm over the following three months without any mechanical force. While literature acknowledges the role of thick gingival or fibrotic tissue in delaying eruption, it is rarely cited as an exclusive cause of impaction [18,19].

The term “soft-tissue impaction” is not consistently defined or recognized in standard orthodontic diagnostic protocols. This case suggests a need to expand our diagnostic framework to include soft-tissue-only impaction as a legitimate clinical category, particularly in patients with sufficient arch space, no pathology, and normal eruption pathways.

The use of a diode laser in this context proved advantageous: it enabled clean, bloodless exposure of the crown with minimal discomfort and no need for general anesthesia. Unlike traditional surgical methods, diode lasers allow precise tissue removal without disturbing adjacent structures, making them well-suited for pediatric patients with mild to moderate impactions [12,14].

Importantly, this conservative management avoided orthodontic brackets, elastics, TADs, or chains. The tooth was not subjected to force; rather, natural eruptive mechanisms resumed once the gingival barrier was removed, supporting the hypothesis that the tooth retained its physiologic eruptive potential. The passive eruption observed, 1 mm at one month and 3 mm at three months, confirms the success of this minimally invasive approach in a carefully selected case.

In addition, the patient’s family history of dental anomalies (including congenitally missing mandibular molars) reflects a possible genetic predisposition that may have contributed to her atypical eruption pattern [3,6]. Mutations in genes such as PAX9, MSX1, and BMP4 have been associated with tooth agenesis, abnormal dental development, and ectopic eruption patterns [20]. These genes play critical roles in odontogenesis and craniofacial development, and their disruption may contribute to eruption disturbances such as impactions [20]. Early screening in similar cases may allow for prompt identification of soft-tissue impactions before more invasive procedures are pursued.

Overall, this case demonstrates that soft-tissue impaction, while under-recognized, is a real and clinically significant condition. Conservative diode laser exposure paired with passive monitoring may offer a successful, patient-centered alternative to traditional traction-based treatment in select cases.

## Conclusions

This case report demonstrates the successful passive eruption of a soft-tissue-impacted maxillary canine following diode laser exposure, without the use of orthodontic traction or bone removal. The results challenge conventional assumptions about the necessity of osseous obstruction in canine impactions and support a conservative, patient-centered approach when anatomical conditions allow. Diode laser exposure, combined with passive space maintenance, should be considered a valid treatment option in well-selected cases, especially in cooperative pediatric patients.

## References

[1] Ericson S, Kurol J (1987). Radiographic examination of ectopically erupting maxillary canines. Am J Orthod Dentofacial Orthop.

[2] McSherry PF (1998). The ectopic maxillary canine: a review. Br J Orthod.

[3] Peck S, Peck L, Kataja M (1994). The palatally displaced canine as a dental anomaly of genetic origin. Angle Orthod.

[4] Jacoby H (1983). The etiology of maxillary canine impactions. Am J Orthod.

[5] Becker A, Chaushu S (2015). Etiology of maxillary canine impaction: a review. Am J Orthod Dentofacial Orthop.

[6] Pirinen S, Arte S, Apajalahti S (1996). Palatal displacement of canine is genetic and related to congenital absence of teeth. J Dent Res.

[7] Coulter J, Richardson A (1997). Normal eruption of the maxillary canine quantified in three dimensions. Eur J Orthod.

[8] Shapira Y, Kuftinec MM (1989). Tooth transpositions--a review of the literature and treatment considerations. Angle Orthod.

[9] Sahim S, Safi-Eddine Z, Aouame A El, Quars F El (2022). Diagnosis and orthodontic management of transposition: a review. OAlib.

[10] D’Amico RM, Bjerklin K, Kurol J, Falahat B (2003). Long-term results of orthodontic treatment of impacted maxillary canines. Angle Orthod.

[11] Yong JSM, Mat Ali UM (2024). Interdisciplinary management of bilateral palatally impacted canines with mini-screws as temporary anchorage devices: a case report. Cureus.

[12] Aoki A, Mizutani K, Taniguchi Y (2024). Current status of Er:YAG laser in periodontal surgery. Jpn Dent Sci Rev.

[13] Shaik KV, Alanazi MIN, Albilasi RM, Albalawi BFA, Alruwaili FA (2021). Lasers in maxillofacial surgery - review of literature. J Pharm Bioallied Sci.

[14] Ishikawa I, Aoki A, Takasaki AA (2004). Potential applications of Erbium:YAG laser in periodontics. J Periodontal Res.

[15] Impellizzeri A, Horodynski M, De Stefano A (2021). Disinclusion of palatally impacted canines with surgical and photobiomodulating action of a diode laser: case series. Appl Sci.

[16] Trybek G, Jaroń A, Gabrysz-Trybek E (2023). Genetic factors of teeth impaction: polymorphic and haplotype variants of PAX9, MSX1, AXIN2, and IRF6 genes. Int J Mol Sci.

[17] Jain S, Debbarma S (2019). Patterns and prevalence of canine anomalies in orthodontic patients. Med Pharm Rep.

[18] Bedoya MM, Park JH (2009). A review of the diagnosis and management of impacted maxillary canines. J Am Dent Assoc.

[19] Kavvadia K, Pepelassi E, Alexandridis C, Arkadopoulou A, Polyzois G, Tossios K (2005). Gingival fibromatosis and significant tooth eruption delay in an 11‐year‐old male: a 30‐month follow‐up. Int J Paediatr Dent.

[20] Zhong X, Liu K, Liu Z, Li C, Chen W (2025). Association between PAX9 or MSX1 gene polymorphism and tooth agenesis risk: a meta-analysis. Open Life Sci.

